# Insights into intrauterine growth restriction based on maternal and umbilical cord blood metabolomics

**DOI:** 10.1038/s41598-021-87323-7

**Published:** 2021-04-09

**Authors:** Georgios Moros, Theodora Boutsikou, Charalambos Fotakis, Zoe Iliodromiti, Rozeta Sokou, Theodora Katsila, Theodoros Xanthos, Nicoletta Iacovidou, Panagiotis Zoumpoulakis

**Affiliations:** 1 Department of Biochemistry & Biotechnology, University of Thessaly, Larisa, Greece; 2 Institute of Chemical Biology, National Hellenic Research Foundation, Athens, Greece; 3 Department of Neonatology, National and Kapodistrian University of Athens, Aretaieio Hospital, Athens, Greece; 4 School of Medicine, European University, Nicosia, Cyprus; 5 Department of Food Science and Technology, University of West Attica, Athens, Greece

**Keywords:** Metabolomics, Intrauterine growth

## Abstract

Intrauterine growth restriction (IUGR) is a fetal adverse condition, ascribed by limited oxygen and nutrient supply from the mother to the fetus. Management of IUGR is an ongoing challenge because of its connection with increased fetal mortality, preterm delivery and postnatal pathologies. Untargeted nuclear magnetic resonance (^1^H NMR) metabolomics was applied in 84 umbilical cord blood and maternal blood samples obtained from 48 IUGR and 36 appropriate for gestational age (AGA) deliveries. Orthogonal projections to latent structures discriminant analysis (OPLS-DA) followed by pathway and enrichment analysis generated classification models and revealed significant metabolites that were associated with altered pathways. A clear association between maternal and cord blood altered metabolomic profile was evidenced in IUGR pregnancies. Increased levels of the amino acids alanine, leucine, valine, isoleucine and phenylalanine were prominent in IUGR pregnancies indicating a connection with impaired amino acid metabolism and transplacental flux. Tryptophan was individually connected with cord blood discrimination while 3-hydroxybutyrate assisted only maternal blood discrimination. Lower glycerol levels in IUGR samples ascribed to imbalance between gluconeogenesis and glycolysis pathways, suggesting poor glycolysis. The elevated levels of branched chain amino acids (leucine, isoleucine and valine) in intrauterine growth restricted pregnancies were linked with increased insulin resistance.

## Introduction

An emerging body of literature indicates that abnormal fetal growth is associated with increased risk of perinatal morbidity and mortality^1–3^. More specifically, intrauterine growth restriction (IUGR) is associated with chronic hypoxia and stress, leading to adverse endocrine axis reprogramming^4,5^. The aforementioned alterations predispose IUGR infants to impaired glucose homeostasis, insulin sensitivity and adipose tissue development, factors that are associated with coronary vascular disease, hypertension and diabetes mellitus in adulthood^6,7^.

IUGR results from the failure of the fetus to reach its intrinsic growth potential, due to maternal, fetal, or placental pathology^8^. The main cause of IUGR is placental insufficiency^9^, causing impaired transfer of nutrients to the fetus due to limited blood flow^10^.

The incidence of IUGR is approximately 8% in total population^11^. Different rates have been recorded in the literature depending on fetal intrinsic factors, as well as prenatal follow-up and optimal perinatal care^12^.

Considering that fetal growth along with gestational age at birth determine neonatal survival, as well as short and long-term complications^1,13^, it is critical to elucidate the underlying pathogenesis of IUGR and the respective implicated metabolic pathways.

Prenatal diagnosis of IUGR mainly relies on abnormal umbilical and uterine Doppler measurements that enable real time estimation of placental/fetal circulation, but do not permit early intervention. Diagnosis is also assisted by an ultrasound-estimated fetal weight below the corresponding 10th percentile for the gestational week^14^.

Thus, it is essential to determine biomarkers framing the time of insult or maternal–fetal metabolic interaction suggesting early intervention. Although several metabolites have been suggested in literature as putative biomarkers for IUGR screening, still none is generally applied in clinical practice^15–18^.

The current study presents a nuclear magnetic resonance (NMR) based untargeted metabolomic approach focusing on IUGR pathology with a twofold aim; (a) to provide explanatory models for IUGR pathogenesis and (b) to identify putative markers of prediction which can be validated at the early stages of pregnancy. Both umbilical cord and maternal blood from well-defined IUGR and appropriate for gestational age (AGA) cases were investigated in one of the biggest sample sizes compared to relevant studies.

## Results

**Figure 1:**
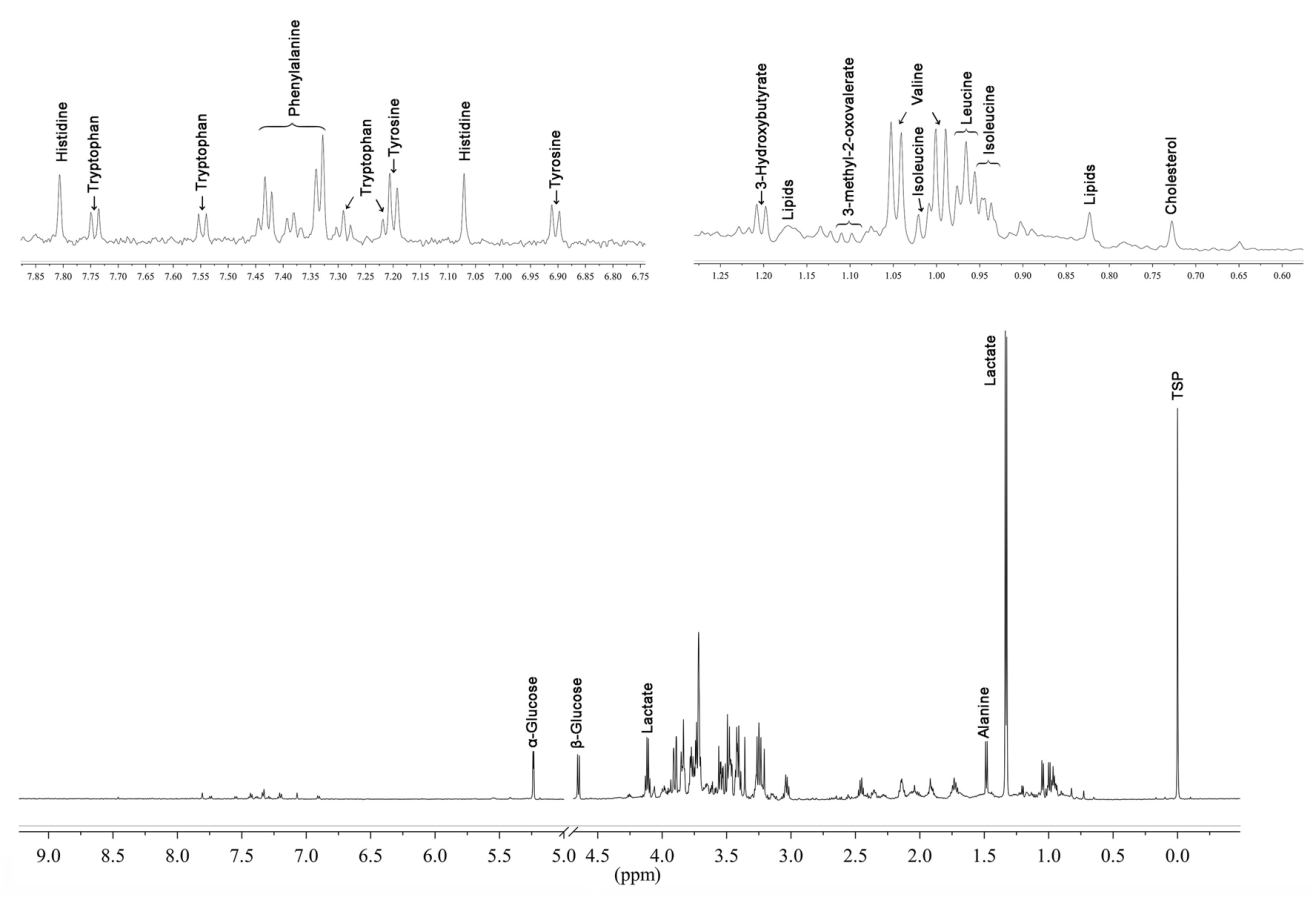
Typical ^1^H-NMR spectrum of serum after methanol extraction following cpmgpr pulse sequence with expanded regions; the region between 3.25 and 4.00 ppm is occupied by sugar resonances. Major metabolites are assigned over the spectrum peaks.

Assignment for 56 individual metabolites excluding the lipid and sugar regions is presented in Supplementary Table S1. Typical ^1^H-NMR spectra with characteristic annotations is presented in Fig. 1.

### Exploratory analysis

Principal component analysis (PCA) on NMR data was implemented in each of the four studied groups (IUGR cord and maternal samples, AGA cord and maternal samples) to assess sample homogeneity and pinpoint outliers. Several characteristics including maternal pathology, smoking habits, gestational age, maternal age, maternal BMI, mode of delivery and gender were examined in each of the produced statistical models. Maternal pathology testing is shown in Supplementary Figs. S1–S4.

Six IUGR participants with gestational diabetes mellitus who presented increased glucose levels and one participant with thrombophilia with a distinct profile were excluded from further investigation from the IUGR group (maternal and cord samples). Furthermore, in umbilical cord blood samples (41 IUGR and 36 AGA), a discrimination trend was observed among IUGR and AGA samples based on the mode of delivery (Supplementary Fig. S5). Samples from vaginal deliveries presented higher lactate levels compared to those from cesarean sections, which necessitates the exclusion of their signals [1.33 (d) and 4.11 (q)] from further data processing. After removing the lactate resonances the impact of labor on blood metabolites was effaced, since no grouping was observed based on the delivery mode.

### Investigation of IUGR-AGA umbilical cord blood samples

**Figure 2:**
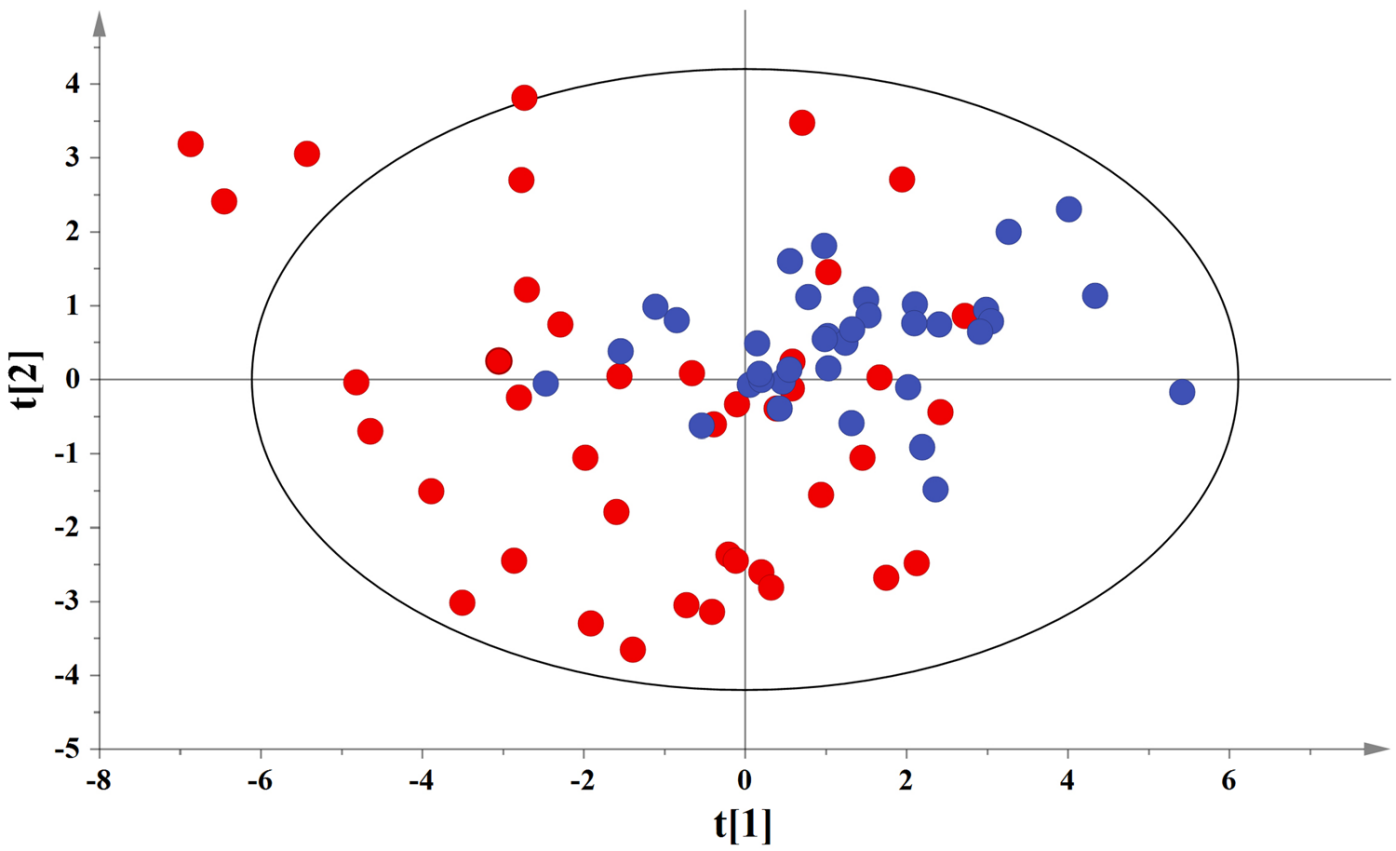
PCA scores plot among the umbilical cord blood samples set. IUGR and AGA samples are represented with red and blue circles, respectively. A = 8, N = 77, R^2^X(cum) = 0.642, Q^2^(cum) = 0.451 for pareto scaling and 95% confidence level. t[1]Eigenvalue:16.6, t[2]Eigenvalue:7.77.

**Figure 3:**
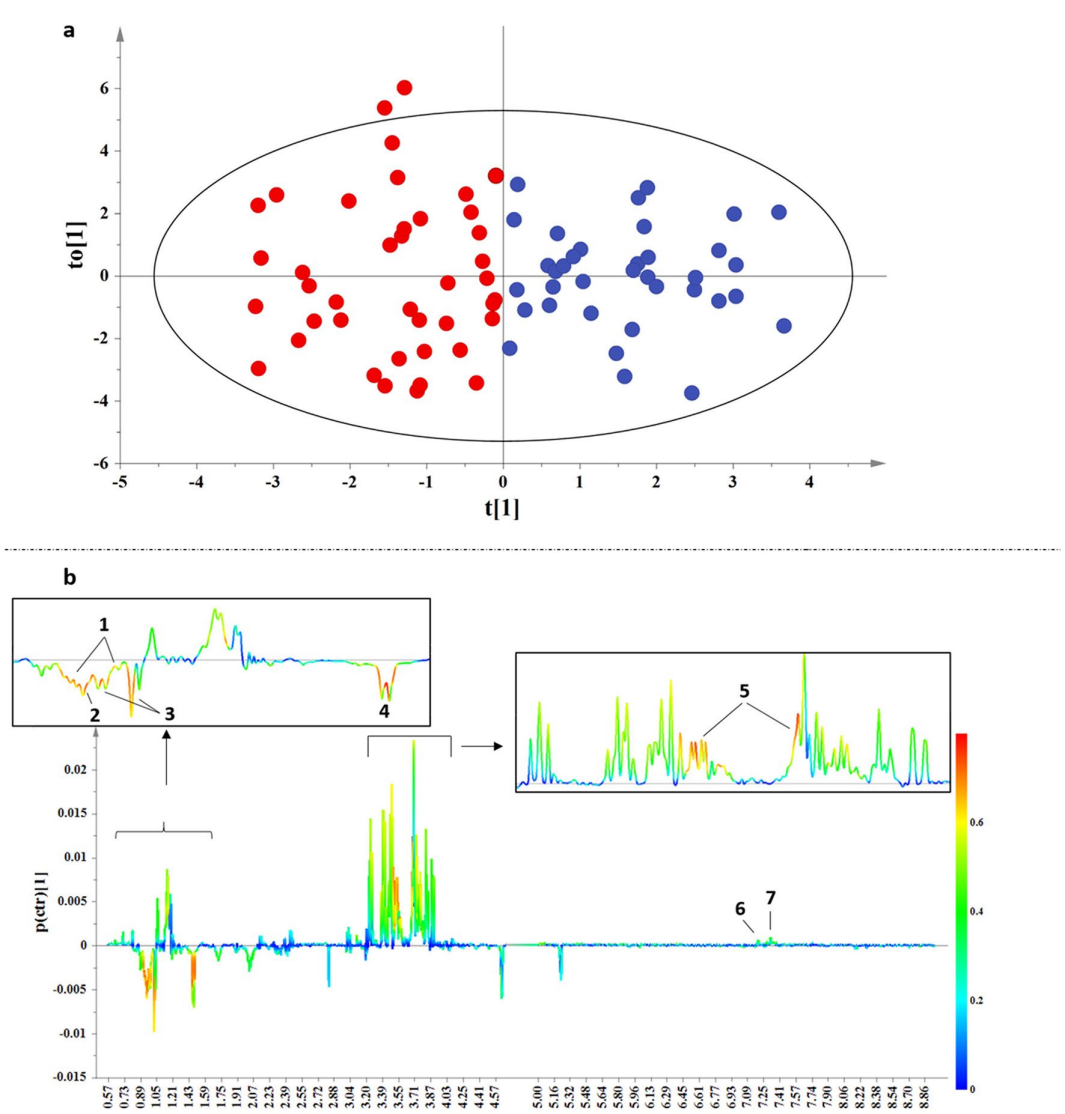
(**a**) OPLS-DA scores for the umbilical cord blood samples, with A = 1 + 1 + 0, N = 77, R^2^X(cum) = 0.279, R^2^Y(cum) = 0.70, Q^2^(cum) = 0.559 for pareto scaling and 95% confidence level. Red circles denote IUGR and blue circles AGA samples. (**b**) Loadings plot demonstrate metabolites responsible for discrimination, 1: Isoleucine, 2: Leucine, 3: Valine, 4: Alanine, 5: Glycerol, 6: Tryptophan, 7: Phenylalanine.

A general overview of the remaining cord blood samples (41 IUGR and 36 AGA) after the removal of lactate and methanol regions is presented in Fig. 2. AGA samples (blue circles) tend to cluster in the first quadrant, while IUGR samples (red circles) are more concentrated in the second, third and fourth quadrants. Figure 3a,b illustrate the OPLS-DA scores and loadings plot for the studied pair, respectively. Permutation testing with receiver operator characteristic (ROC) curves validated the OPLS-DA discrimination (Supplementary Fig. S6a).

As depicted in Fig. 3b, IUGR umbilical cord blood samples are characterized by higher levels of the branched chain amino acids (BCAAs) leucine, isoleucine and valine and the non-essential amino acid alanine. On the other hand, AGA samples are characterized by higher levels of the essential aminoacids phenylalanine and tryptophan. Moreover, glycerol is altered between the two test-groups, being elevated in AGA samples. Biomarker analysis (Supplementary Fig. S7) confirmed the above findings for the suggested metabolites between the two groups revealing a high Area Under the Receiver Operating Characteristic curve (AUROC).

### Investigation of IUGR-AGA maternal blood samples

**Figure 4:**
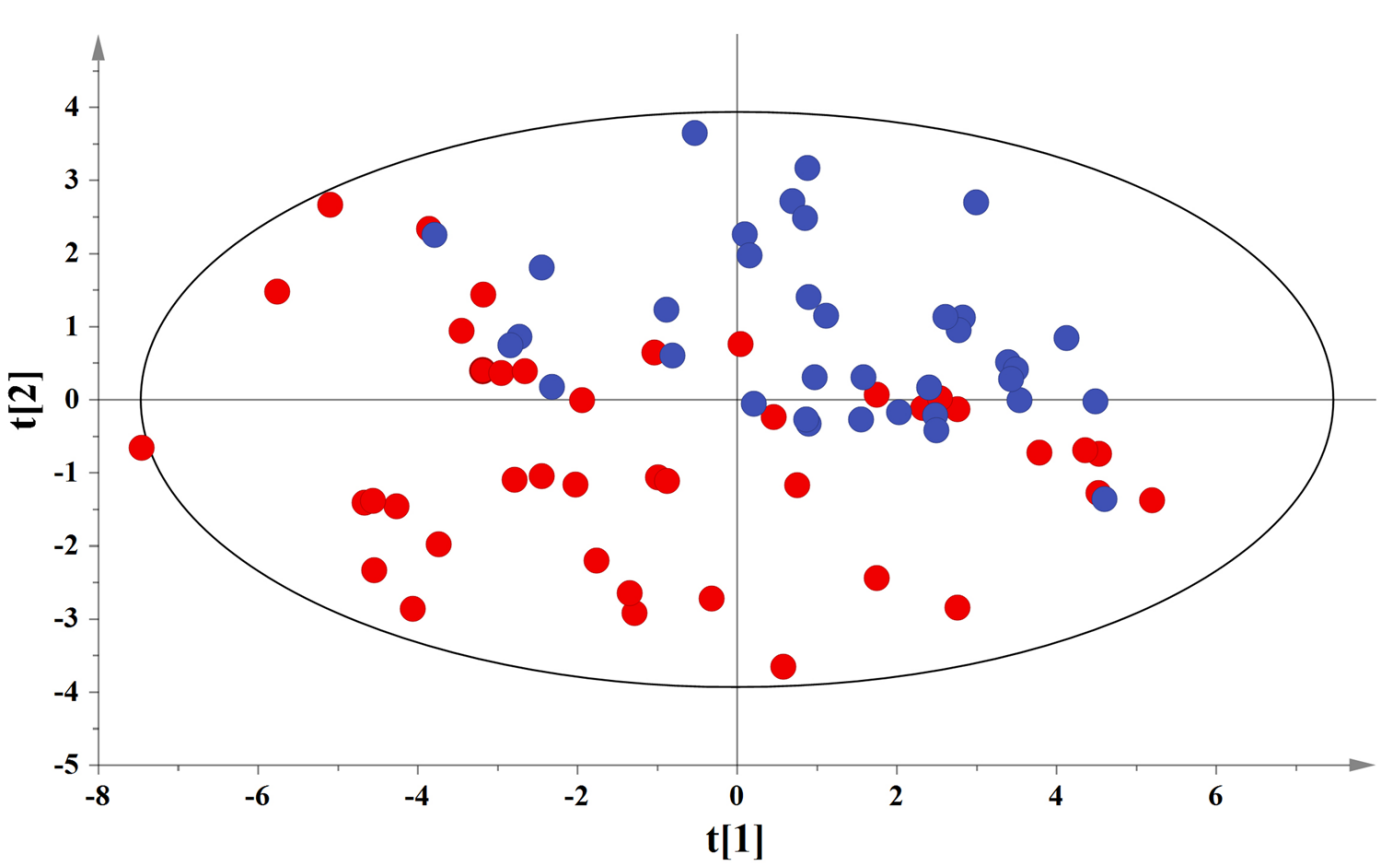
PCA scores plot among the maternal blood samples set. IUGR and AGA samples are represented with red and blue circles, respectively. A = 8, N = 77, R^2^X(cum) = 0.666, Q^2^(cum) = 0.506 for pareto scaling and 95% confidence level. t[1] Eigenvalue:22.7, t[2] Eigenvalue:6.3.

**Figure 5:**
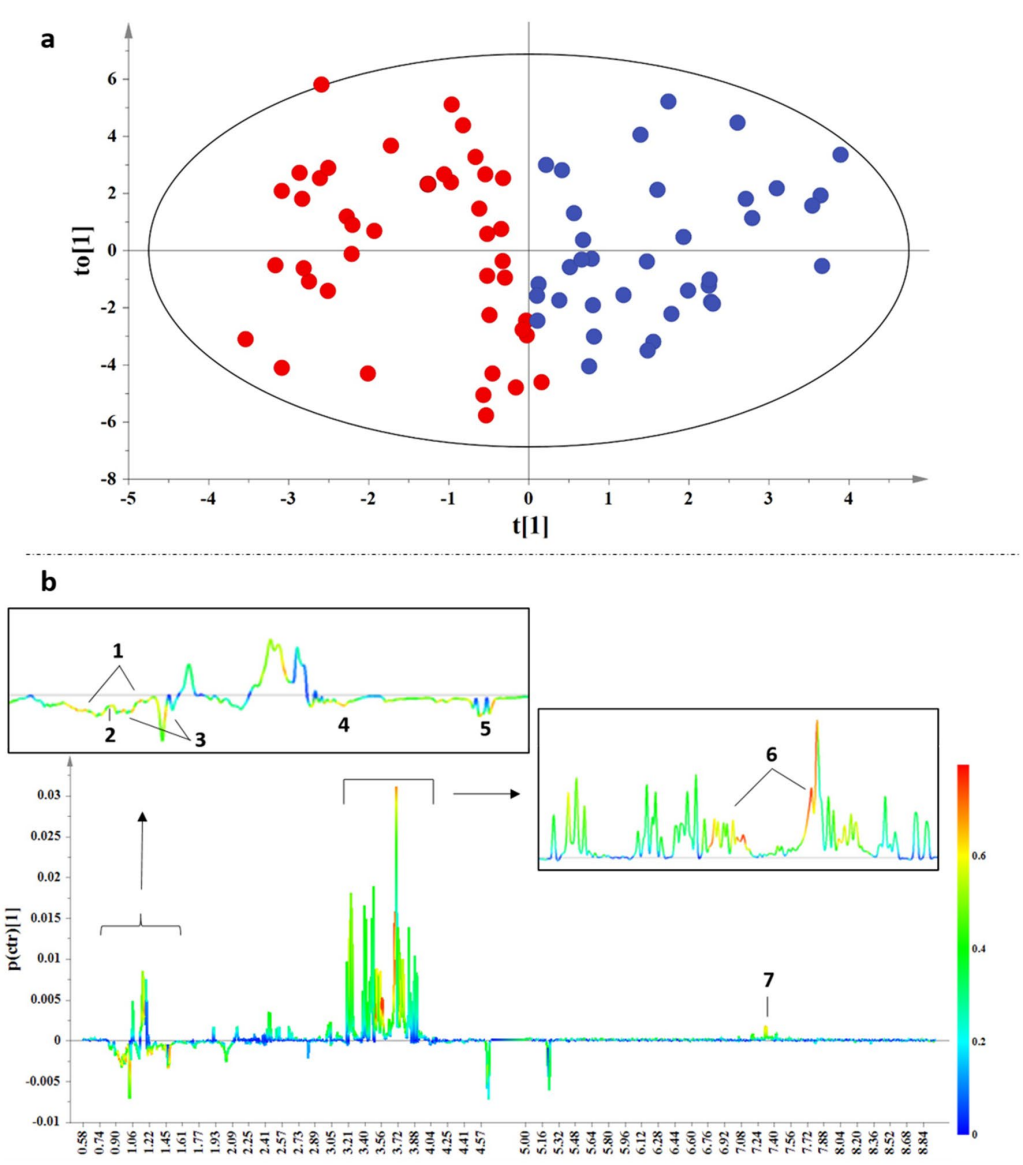
(**a**) OPLS-DA scores plot for maternal samples with A = 1 + 1 + 0, N = 77, R^2^X(cum) = 0.369, R^2^Y(cum) = 0.65, Q^2^(cum) = 0.566 for pareto scaling and 95% confidence level. IUGR are represented with red circles and AGA correspond to blue circles. (**b**) Loadings plot demonstrate the metabolites responsible for discrimination, 1: Isoleucine, 2: Leucine, 3: Valine, 4: 3-Hydroxybutyrate, 5: Alanine, 6: Glycerol, 7: Phenylalanine.

A similar data analysis process was followed among maternal samples. Figure 4 illustrates a trend of clustering among the second principle component of the PCA. More specifically IUGR samples (red circles) create a cluster among the 3rd and 4th quadrants while AGA samples (blue circles) among the 1st and 2nd quadrants. Figure 5a,b illustrate the OPLS-DA scores and loadings plot for the studied pair, respectively. Validation tests are presented in Supplementary Fig. S6b.

The metabolites assigned to the discriminatory bins of the loadings plot (Fig. 5b) along with significant AUROC (Supplementary Fig. S8), correspond to the essential amino acids leucine, isoleucine, valine and phenylalanine, the non-essential amino acid alanine and the organic metabolites namely glycerol and 3-hydroxybutyrate.

### Significant metabolites and altered pathways

Table 1 presents the AUROCs for the discriminatory metabolites between test-groups. Univariate testing is presented in Table 2.

**Table 1:** Comparison of AUROCs for discriminatory metabolites among umbilical cord blood and maternal samples.

Altered metabolite	AUROC	
	Umbilical cord blood	Maternal blood
Alanine	**0.871**	0.792
Leucine	0.816	0.773
Isoleucine	0.795	**0.812**
Valine	0.785	0.786
Phenylalanine	0.779	0.75
Glycerol	0.835	0.751
Tryptophan	0.751	Non-significant
3-Hydroxybutyrate	Non-significant	0.774

Values in bold refer to metabolites presenting the higher AUROC.

**Table 2:** P-values and fold changes for significant metabolites among umbilical and maternal blood samples.

Altered metabolite	Umbilical cord blood		Maternal blood	
	p-value	Fold changes^a^	p-value	Fold changes^a^
Alanine	6.47 × 10^–9^	− 0.47	3.76 × 10^–6^	− 0.54
Leucine	1.31 × 10^–6^	− 0.42	5.81 × 10^–5^	− 0.32
Isoleucine	6.39 × 10^–6^	− 0.44	8.76 × 10^–7^	− 1.07
Valine	4.77 × 10^–6^	− 0.47	7.18 × 10^–6^	− 0.48
Phenylalanine	3.05 × 10^–5^	2.42	9.73 × 10^–5^	0.41
Glycerol	1.59 × 10^–7^	0.45	1.84 × 10^–4^	0.26
Tryptophan	7.52 × 10^–5^	3.17	Not-significant	Not-significant
3-Hydroxybutyrate	Non-significant	Non-significant	2.58 × 10^–5^	-0.50

^a^Positive values indicate metabolites determined in higher concentration in the AGA group whereas negative values indicate metabolites determined in higher concentration in the IUGR group.

Pearson correlation testing between the peak integral areas of the remaining lipids at 0.82 and 1.16 ppm with the BCAAs and the 3-hydroxybutyrate areas for 10 representative samples of each group (umbilical and maternal) probed to no significant correlation (weak to moderate values of the correlation coefficient) (Supplementary Tables S2 and S3). Interestingly, 6 out of 8 proposed metabolites were prominent in both umbilical cord blood and maternal group discrimination with high degree of statistical significance (AUROC > 0.75 and p-value < 0.05). In particular, higher levels were recorded for the branched chain amino acids BCAAs (leucine, isoleucine and valine) and alanine in IUGR groups, in contrast to the increased phenylalanine and glycerol levels in the AGA groups.

On the other hand, tryptophan contributed only to the umbilical cord blood discrimination, whereas 3-hydroxybutyrate only to the maternal blood discrimination. Tryptophan showed increased levels in AGA umbilical cord blood samples and 3-hydroxybutyrate showed increased levels in IUGR maternal blood samples. Alanine is the most significant metabolite (AUROC = 0.871) for cord blood discrimination and isoleucine (AUROC = 0.812) for maternal discrimination.

Enrichment analysis provided the deregulated pathways due to the IUGR pathology. Supplementary Tables S4 and S5 present the three most probably affected pathways in umbilical cord blood and maternal blood sample pairs respectively. Pathways with the higher number of hits and a p value lower than 0.05 (statistically significant) were selected as the candidate key pathways. The most significantly affected metabolic pathway in both pairs reflects the Valine, Leucine and Isoleucine Degradation.

## Discussion

A similar metabolic pattern was evident for the first time between maternal and umbilical cord blood in IUGR pregnancies. This finding may indicate common mechanisms underlying the IUGR condition which connected between mothers and fetuses and can pave the way for the identification of putative biomarkers in maternal blood targeting early IUGR screening.

Controversial information is evidenced in the literature concerning candidate metabolites for IUGR-AGA discrimination, with abnormal amino acid metabolism, glucose intolerance and limited insulin resistance being an important finding^19^.

IUGR off springs have altered protein expression profiles^20^ which may result in a reduced utilization rate of the BCAAs and alanine, leading to a consequent increase of their levels. This increase could also be explained by the observation that IUGR fetuses experience the same metabolic derangements with certain diseases, with urea cycle defects being the most relevant^21^.

Starving conditions promote protein degradation leading to the consequent increase of BCAAs. These are degraded in tissues, other than liver, yielding reduced NADH and FADH_2_ which facilitate the energy production^22^. IUGR is linked to reduced oxygen and nutrients supply through the placenta^23^ which could partly explain the observed BCAAs increase. Nutrient deficiency has also been proposed to describe the up-regulation of valine and isoleucine in the IUGR vs AGA groups^16^.

Moreover, increased levels of BCAAs have been associated with insulin resistance and future type 2 diabetes^24–26^. Dessi et al*.*^18^ suggested that IUGR fetuses stimulate a number of adaptive mechanisms in order to save glucose and promote nutrition of the vital organs, resulting in down-regulation of insulin secretion. Herein, the observed increase of BCAAs in the IUGR group could be associated with insulin resistance of the fetus. Glucose intolerance or limited insulin adequacy in IUGR, may account for altered tricarboxylic acid (TCA) metabolic intermediates, such as alanine and leucine^27^.

In a previous type 2 diabetes metabolomic study^26^, the branched-chain keto acid (BCKA) 3-methyl-2-oxovalerate, has been identified as one of the strongest predictors of impaired fasting glucose (IFG). This BCKA arises from the incomplete catabolism of BCAAs^26,28^ which primarily occurs in the mitochondria. High levels of the corresponding metabolite are also linked with the metabolic disorder of maple syrup urine disease that is caused by the deficiency of the branched-chain alpha-keto acid dehydrogenase complex, leading to the accumulation of BCAAs^28^.

The glucose-alanine cycle describes the synthesis of glucose from alanine in the liver and the transport of glucose back to the muscles. Alanine serves as a precursor or regulator of glucose metabolism that is of critical importance in the nutrient-deprived IUGR, thus explaining the increased alanine levels found in the IUGR group^22^. Higher alanine levels were also found for IUGR having a birth weight between 3rd and 10th percentile^29^.

In our research, lower glycerol levels were observed in the IUGR group, signifying glycerol deprivation. Gluconeogenesis is a pathway describing glucose synthesis from lactate, pyruvate, glycerol and glucogenic amino acids^30^. In the liver, glycerol may be converted into glucose, meeting the required energy needs for cellular metabolism^31^. Glycolysis, a compensatory pathway that breakdowns glucose, is deregulated in conditions of limited insulin adequacy or resistance^32^. IUGR infants have insulin deficiency^18^ which partly explains poor glycolysis together with a continuous conversion of glycerol to glucose through gluconeogenesis. Also, lower glycerol levels in the IUGR groups could indicate an imbalance between triacylglycerol breakdown and re-synthesis during several metabolic reactions in a continuous flux between adipose tissue and liver.

In pregnant women with glucose intolerance, ketone body formation is favored against carbohydrate consumption^33^, resulting in the accumulation of 3-hydroxybutyrate. Furthermore, the observed increase of the ketogenic essential amino acids leucine and isoleucine, may also account for increased 3-hydroxybutyrate synthesis^33^. The metabolomic study of Powel et al*.*^34^ suggested 3-hydroxybutyrate is a predictor of IUGR complication in maternal serum as well, however, with low predictive power.

Different brain serotonin synthesis has been associated with altered tryptophan levels^16^. Cosmi et al*.*^35^ in line with our observations indicated tryptophan as a candidate marker of IUGR pathology, but reported findings are different to our results regarding our observed up-regulation of valine and isoleucine and the down-regulation of phenylalanine. Of note, such outcomes refer to twin neonates and are deprived by limited sampling.

Paolini et al*.*^36^ found a lower fetal/maternal enrichment ratio for phenylalanine and leucine in IUGR compared to normal pregnancies, suggesting an impaired transplacental flux. An altered placental metabolism or transport alongside with a higher catabolic condition has been also suggested to explain the lower phenylalanine levels in the IUGR samples^16,37^. The fetomaternal ratio for the essential amino acids is reduced in cases of small for gestational age (SGA) compared to AGA, as a result of fetal hypoxemia^38^. Finally, increased levels of BCAAs and alanine in the maternal blood of IUGR vs AGA groups, is possibly explained due to the altered adaptation mechanisms of IUGR pregnancy with incomplete hormone production^38,39^.

## Conclusions

In our study we interpreted the generated metabolomic data and attempted to correlate a fetal adverse condition with alterations in the metabolic profile as putative biomarkers. A similar metabolic profile was evident between maternal and umbilical cord blood in IUGR pregnancies, with seven out of 56 identified metabolites being responsible for discrimination. As an exception, tryptophan contributed only to cord blood discrimination while 3-hydroxybutyrate only to maternal blood discrimination.

Higher levels of the BCAAs, together with alanine were observed in the IUGR groups, in contrast to increased phenylalanine and glycerol levels for the AGA groups, indicating the crucial roles of amino acid metabolism, insulin resistance and glycolysis pathway. The elevated levels of BCAAs in intrauterine growth restricted pregnancies were linked with increased insulin resistance. Although a number of clinical studies have facilitated metabolomics to elaborate on the IUGR condition, biomarkers that determine this condition early on, are still lacking. From our findings, the role of previously reported metabolites has been strengthened together with the significance of 3-hydroxybutyrate in IUGR maternal blood. The proposed metabolic signatures have to be validated at an earlier stage of pregnancy in order to obtain clinical impact as diagnostic and prognostic biomarkers for IUGR.

## Methods

### Ethical statement

An approval (No. EE-2/26/06-06-2017) from the Aretaieio Hospital Review Board along with the Ethics committee was received before the experiments were started. All experiments were performed in accordance relevant guidelines and regulations. Signed informed consent was obtained from the participating mothers prior to enrollment.

### Patient recruitment

The study comprised 84 parturients giving consecutively birth to 48 IUGR (≤ 10th customized centile) and 36 AGA (between 10 and 90th customized centile) full-term infants (Supplementary Table S6). Among IUGR pregnancies, 6 were complicated by gestational diabetes mellitus (GDM) and 1 by thrombophilia. Median gestational age for IUGR neonates was 273 (± 7 days) and median birth weight was 2610 (± 263 g). Median gestational age for AGA neonates was 278 (± 6 days) and median birth weight was 3405 (± 273 g). Diagnostic testing is presented in Supplementary Methods.

### Sample collection and pretreatment

Mixed arteriovenous blood was collected from the doubly clamped umbilical cord, reflecting fetal blood. Maternal blood was collected either at the first stage of labor in cases of vaginal delivery or before receiving anesthesia in cases of caesarean section, in fasting conditions; time between last food intake and sampling were at least 8 h. Samples allowed to clot at room temperature for 1 h and centrifuged at 1500×*g* for 10 min. Serum was collected in eppendorf tubes and transferred to − 80 °C till sample preparation.

Serum samples were thawed at room temperature and extracted according to Nagana Gowda et al*.*^40^ protocol. In particular, 250 μL serum samples were extracted with 500 μL methanol (1:2 v/v), vortexed and placed at − 20 °C for 20 min. Samples were centrifuged (11,000 rpm/ 4 °C) for 20 min to allow protein parts to precipitate. Supernatants placed to new eppendorf tubes and evaporated to dryness. Samples were reconstituted to 400 μL D_2_O and 150 μL phosphate buffer (0.2 M, Na_2_HPO_4_ 2H_2_O and NaH_2_PO_4_, pH = 7.0). Then, 500 μL of each mixture with 50 μL of internal standard trimethylsilyl propionic acid sodium salt (TSP) (2.75 mM) were transferred to 5 mm NMR tubes for ^1^H-NMR analysis.

### ^1^H-NMR spectroscopy

All ^1^H-NMR spectra were acquired using a Varian 600 MHz spectrometer equipped with a triple resonance probe (^1^H, ^13^C, ^15^N), at room temperature (25 °C). The CPMG pulse sequence following water suppression was applied in all experiments since it provided better spectra in terms of S/N ratio compared to 1DNOE pulse sequence. In total, 128 transients were collected with 64 K data points and a relaxation delay of 5 s. Receiver gain was auto set 60 for all acquisitions. All ^1^H NMR spectra were referenced at TSP chemical shift (0.00 ppm) and processed at 0.3 exponential line broadening.

### Data handling and metabolite assignment

All ^1^H-NMR spectra were preprocessed with MestreNova (v. 10.1) software. Manual phase correction, automatic baseline correction and sinc apodization were applied to improve spectra resolution. Total area normalization and binning of 0.001 ppm were selected. Superimposed spectrum was constructed and peaks were manually aligned. The water region (4.68–5.00 ppm) was excluded together with the peak at 3.36 ppm attributed to methanol from protein precipitation procedure.

Peak assignment and metabolite identification were performed using 2D NMR experiments (gCOSY, zTOCSY, gHSQCad and gHMBCad) on a representative pool sample. Assignment of spectral peaks was assisted by Chenomx database (Chenomx Suite 7.6, Chenomx, Edmonton, Alberta, Canada), an in-house software (Metaboneer)^41^ and the online NMR databases HMDB^42^ and BMRB^43^. The metabolites 3-hydroxybutyrate, isoleucine as well as the 3-methyl-2-oxovalerate that presented low signal to noise ratio were further elucidated by spiking the reference pool sample and performing 2D gHMBCad NMR experiment (Supplementary Figs. S9, S10). To a step further we utilized two specialized software, the online platform Bayesil^44^ and ASICS^45^. Each software proposed a number of metabolites for every sample and provided quantitative results (Supplementary Datasets 1 and 2).

### Statistical analysis and identification of discriminant metabolites

Multivariate statistical analysis was employed to aligned spectra after Pareto scaling, using SIMCA software (v. 14.0, Umetrics, Umea, Sweden). At first, PCA was applied to provide a general insight (trends, clusters, outliers) of samples. OPLS-DA was applied next, to generate classification models. Model performance assessed through the R^2^Y (goodness of fit) and Q^2^ (goodness of prediction) values. Validation of discrimination was made through response permutation testing (999 permutations), analysis of variance (CV-ANOVA) and extraction of ROC curves.

Color coded loadings plots (s-line) attributed certain metabolites responsible for the discrimination pattern. MetaboAnalyst 4.0^46^ was applied next to find significant metabolites discriminating umbilical cord blood and maternal groups. Biomarker analysis between IUGR-AGA pairs revealed candidate biomarkers (a cut off value of 0.75 for AUROC was selected) and used for enrichment analysis to provide altered metabolic pathways.

## Supplementary Information

Supplementary Information 1.

Supplementary Information 2.

Supplementary Information 3.

## Data Availability

Restrictions apply to the availability of raw data, since were used under license for the current study. Data are however available from the corresponding authors upon justified request.
