# Supplementary material for: Insights into intrauterine growth restriction based on maternal and umbilical cord blood metabolomics
Source: Sci Rep. 2021 Apr 9;11:7824. doi: 10.1038/s41598-021-87323-7 (PMC8035183; doi:10.1038/s41598-021-87323-7)
Supplement: Supplementary file 1 — Supplementary Information 1. [file 41598_2021_87323_MOESM1_ESM.pdf]

Supplementary information

Insights into intrauterine growth restriction based on maternal and umbilical cord blood metabolomics

Georgios Moros[1,2,+], Theodora Boutsikou[3,+], Charalambos Fotakis[2], Zoe Iliodromiti[3], Rozeta Sokou[3], Theodora Katsila[2], Theodoros Xanthos[4], Nicoletta Iacovidou[3,*], Panagiotis Zoumpoulakis[2,5*]

Supplementary Methods

Diagnostic testing. Gestational age was determined by a combined estimation of the first day of the mother's last menstrual period and a first trimester ultrasound scan. The customized centile for each pregnancy was calculated using the computer generated program AUDIPOG that required significant determinants of birth weight (maternal height and booking weight, parity, gestational age and gender) to adjust the normal centile limits for birth weight. All neonates had one- and five-minute Apgar score ≥8 and presented with normal cord blood arterial pH.

Umbilical artery blood flow patterns and resistance indices (RI) were documented, with an umbilical artery RI outside the normal range depicting a pathologic flow pattern. All IUGR cases presented with impaired blood flow. However, there were no cases of absent end-diastolic flow in the umbilical arteries. Amniotic fluid and placental weight were reduced in all IUGR cases, a finding indicating placental insufficiency.

Supplementary Figures

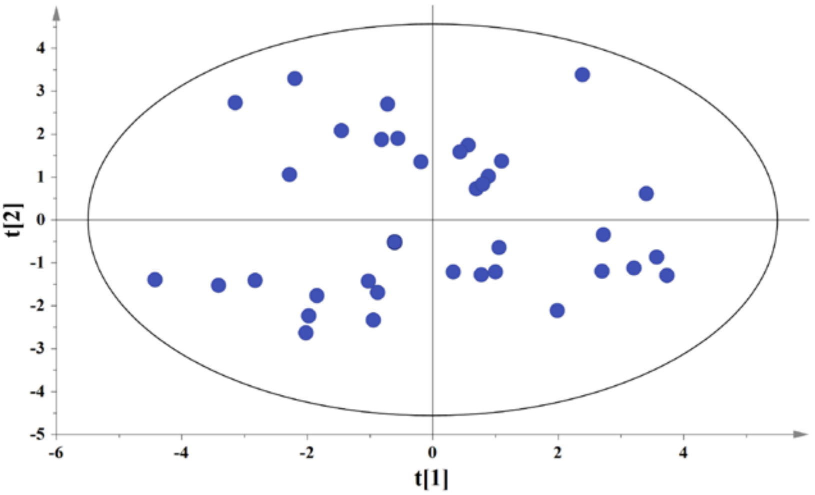

Supplementary Figure S1. PCA scores plot among AGA umbilical cord samples. A=5, N=36, $R^2X(cum)=0.631$, $Q^2(cum)=0.435$ for pareto scaling and 95% confidence level. t[1] Eigenvalue:7.90, t[2] Eigenvalue:5.45.

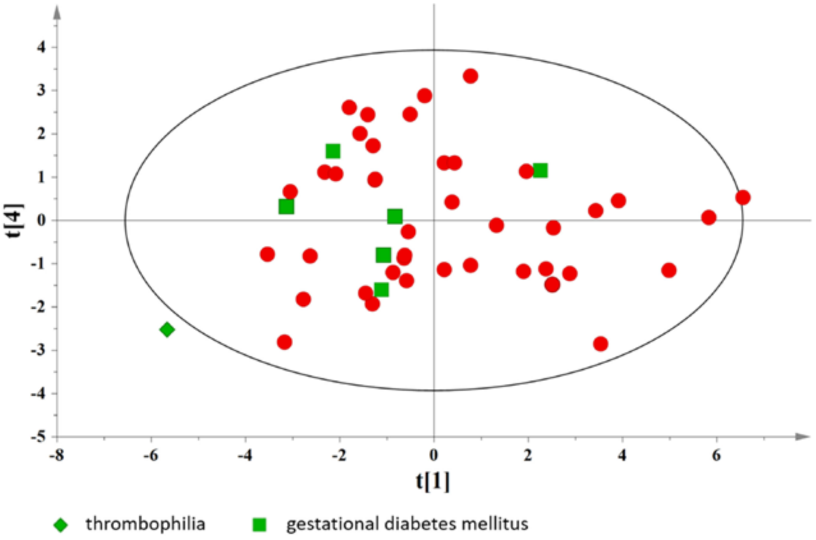

Supplementary Figure S2. PCA scores plot among IUGR umbilical cord samples. Samples with maternal pathology are represented with green color: squares represent samples with gestational diabetes mellitus and rhombus represent a sample with thrombophilia. A=6, N=48, $R^2X(cum)=0.649$, $Q^2(cum)=0.435$ for pareto scaling and 95% confidence level. t[1] Eigenvalue:11, t[2] Eigenvalue:6.32, t[3] Eigenvalue: 4.64, t[4] Eigenvalue: 3.98.

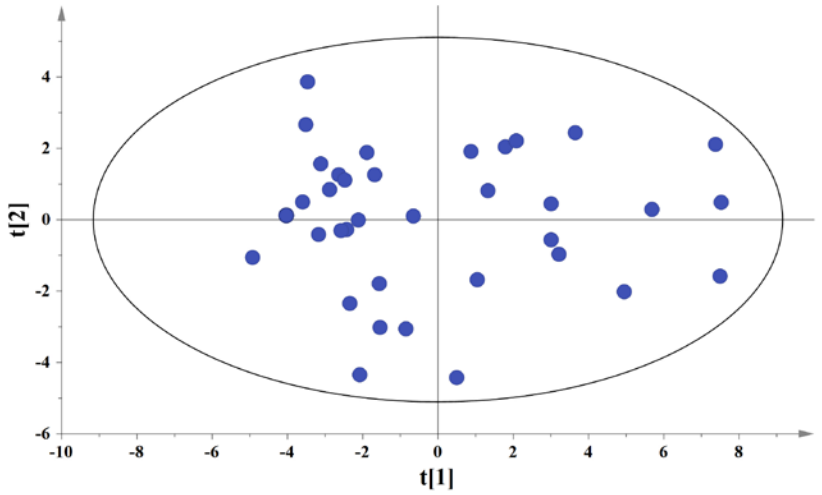

Supplementary Figure S3. PCA scores plot among AGA maternal samples. A=4, N=36, R^2X(cum)=0.638, Q^2(cum)=0.476 for pareto scaling and 95% confidence level. t[1] Eigenvalue:13.4, t[2] Eigenvalue:4.16, t[3] Eigenvalue:3.02.

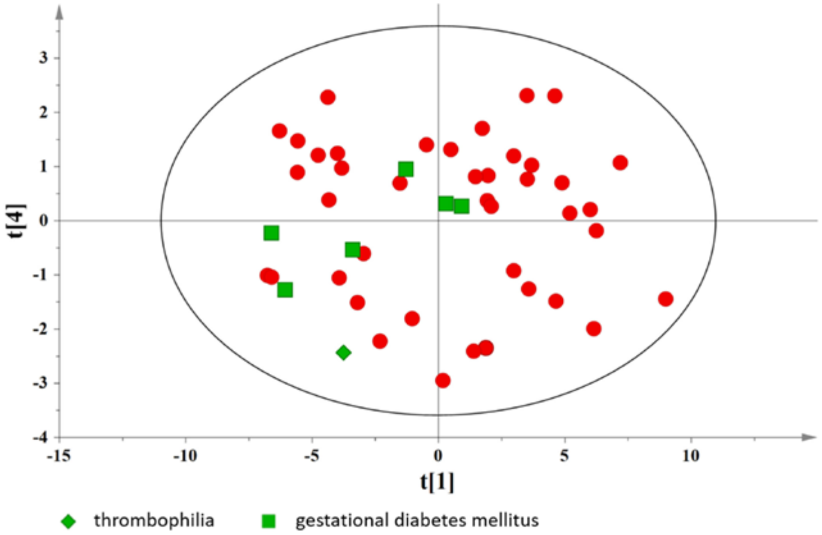

Supplementary Figure S4. PCA scores plot among IUGR maternal samples. Samples with pathology are represented with green color: squares represent samples with gestational diabetes mellitus and rhombus represent sample with thrombophilia. A=5, N=48, R^2X(cum)=0.678, Q^2(cum)=0.543 for pareto scaling and 95% confidence level. t[1] Eigenvalue:21.2, t[2] Eigenvalue:3.89, t[3] Eigenvalue: 3.17.

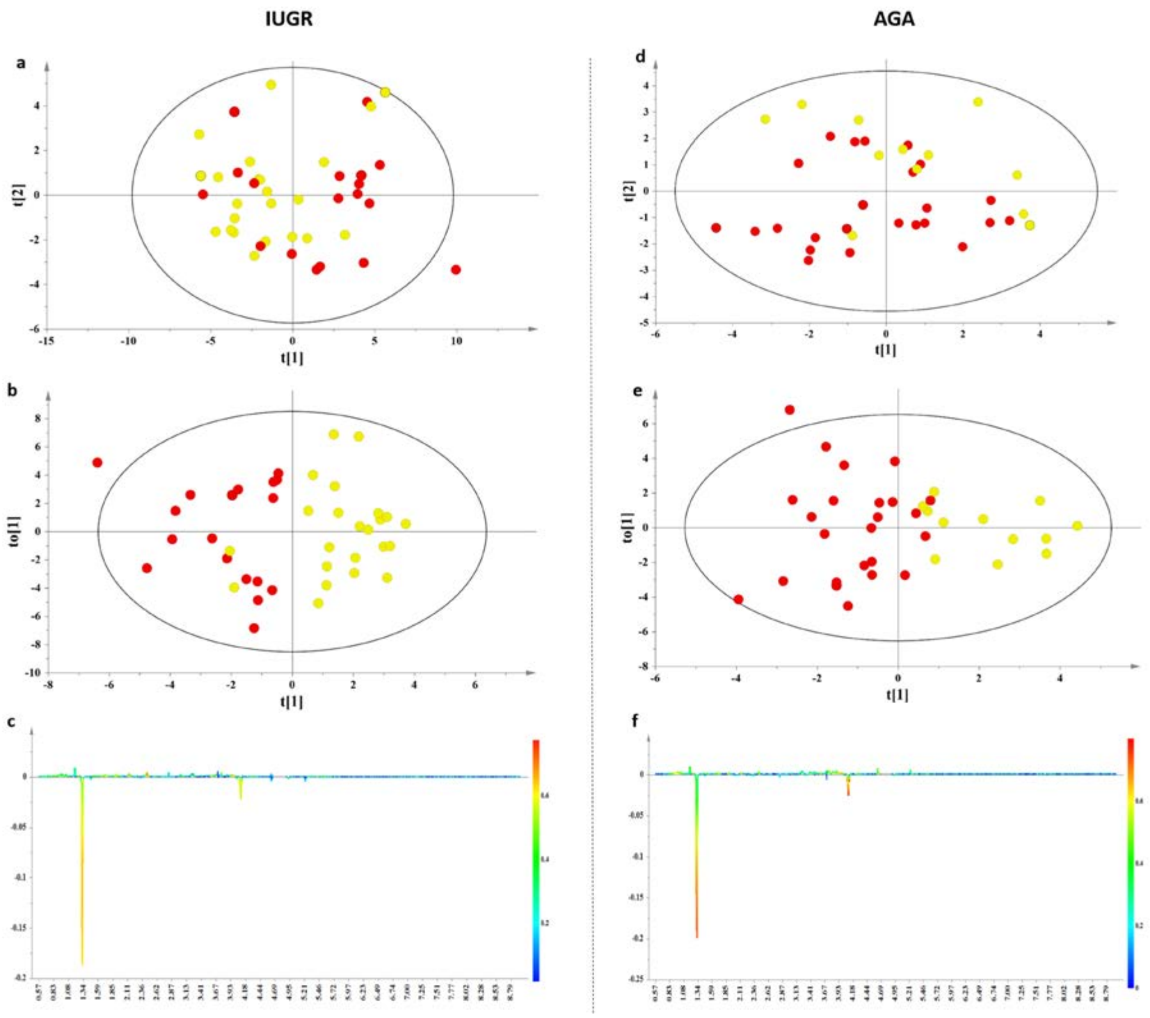

Supplementary Figure S5. Multivariate statistical analysis on umbilical cord blood samples showing the effect of Vaginal (red circles) and Caesarean (yellow circles) delivery on samples. **(a)** PCA scores plot over IUGR umbilical cord blood samples [A=4, N=41, $R^2X(cum)=0.626$, $Q^2(cum)=0.485$, t[1] Eigenvalue:14.8, t[2] Eigenvalue:5.04]; **(b)** OPLS-DA scores plot over IUGR umbilical cord blood samples [A=1+1+0, N=41, $R^2X(cum)=0.423$, $R^2Y(cum)=0.607$ $Q^2(cum)=0.256$]; **(c)** s-line loadings plot for IUGR OPLS-DA model; **(d)** PCA scores plot over AGA umbilical cord blood samples [A=5, N=36, $R^2X(cum)=0.631$, $Q^2(cum)=0.435$, t[1] Eigenvalue:7.90, t[2] Eigenvalue:5.45]; **(e)** OPLS-DA scores plot over AGA umbilical cord blood samples [A=1+1+0, N=36, $R^2X(cum)=0.365$, $R^2Y(cum)=0.629$ $Q^2(cum)=0.278$]; **(f)** s-line loadings plot for AGA OPLS-DA model.

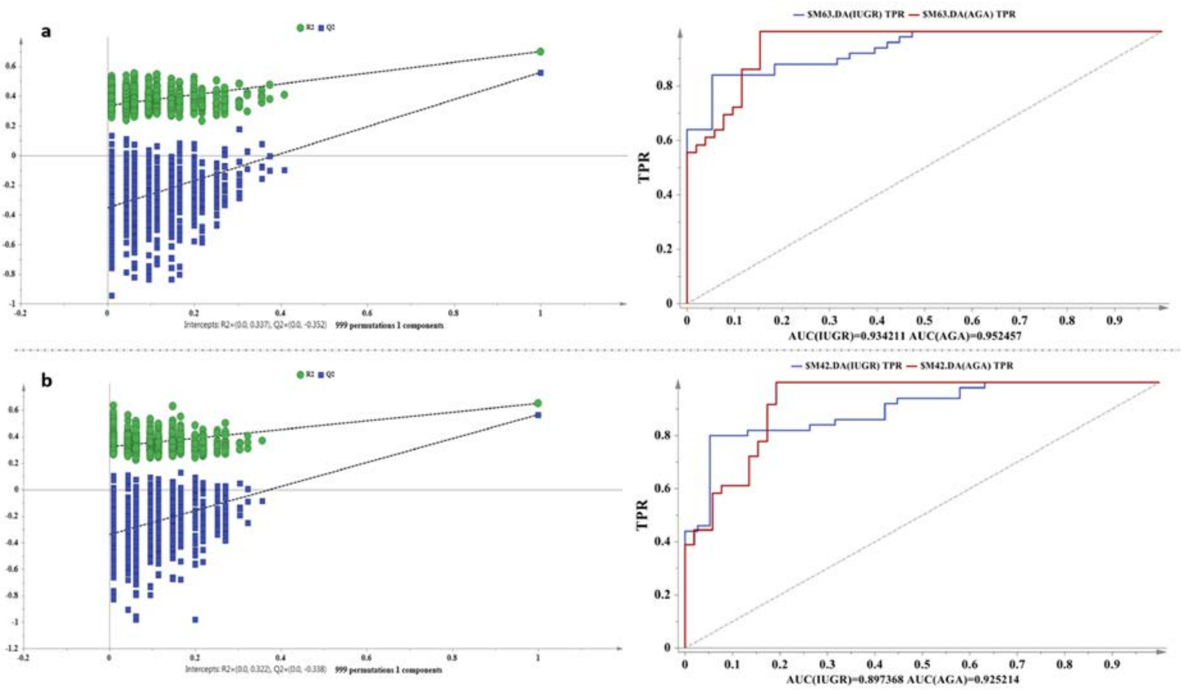

Supplementary Figure S6. (a) Permutation testing with ROC curves for IUGR-AGA umbilical cord blood discriminant analysis, **(b)** Permutation testing with ROC curves for IUGR-AGA maternal discriminant analysis. Considerable p values observed in both discriminations.

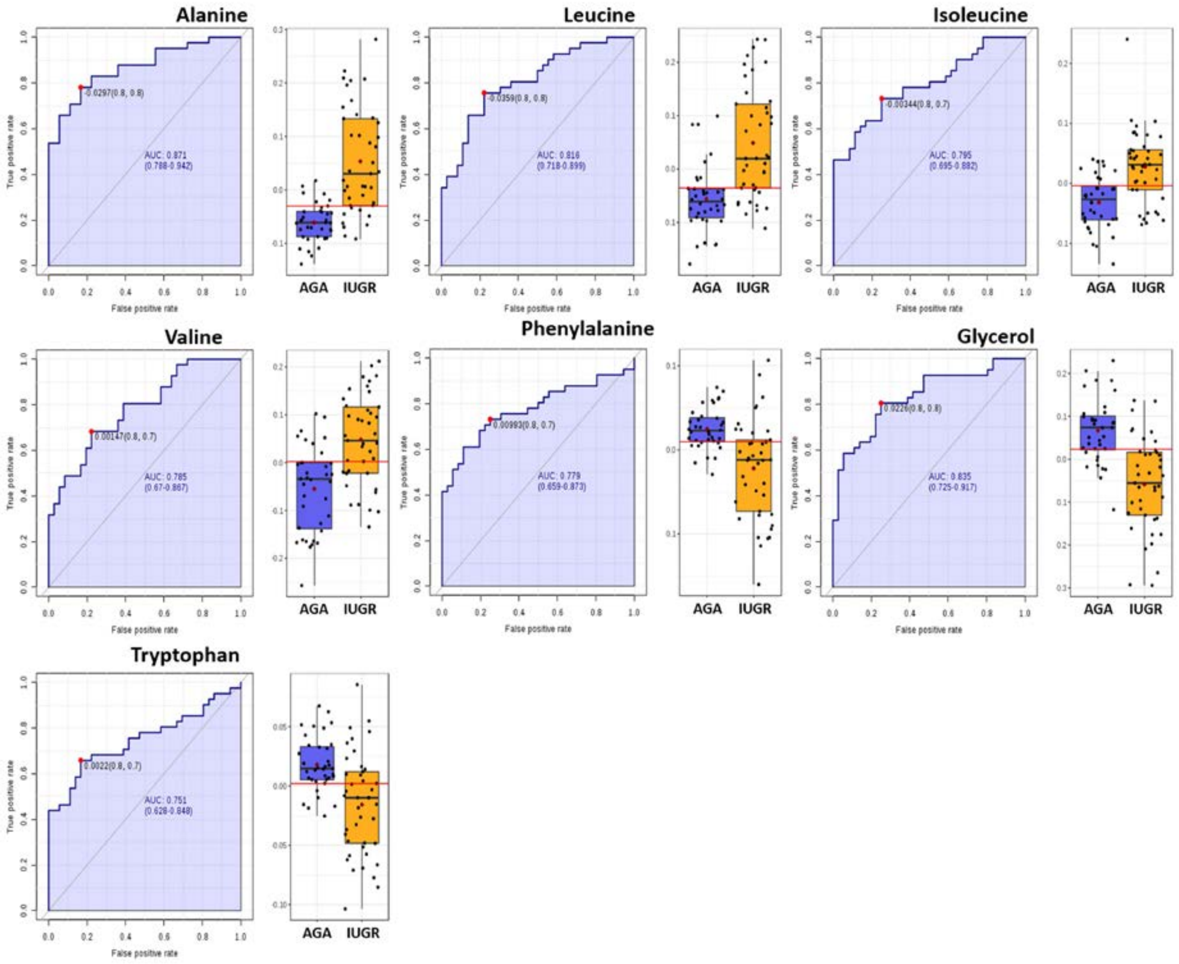

Supplementary Figure S7. ROC curves and box plots for important metabolites concerning the IUGR-AGA umbilical cord blood discrimination.

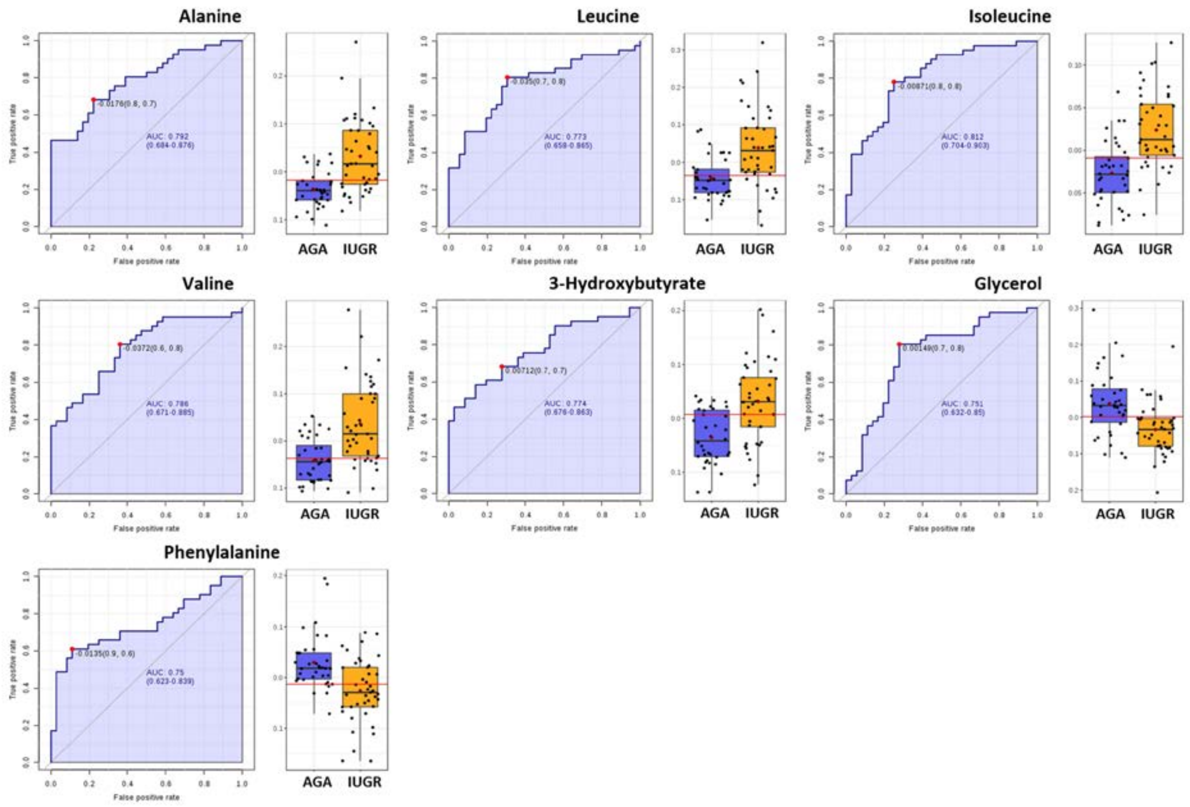

Supplementary Figure S8. ROC curves and box plots for significant metabolites concerning the IUGR-AGA maternal blood discrimination.

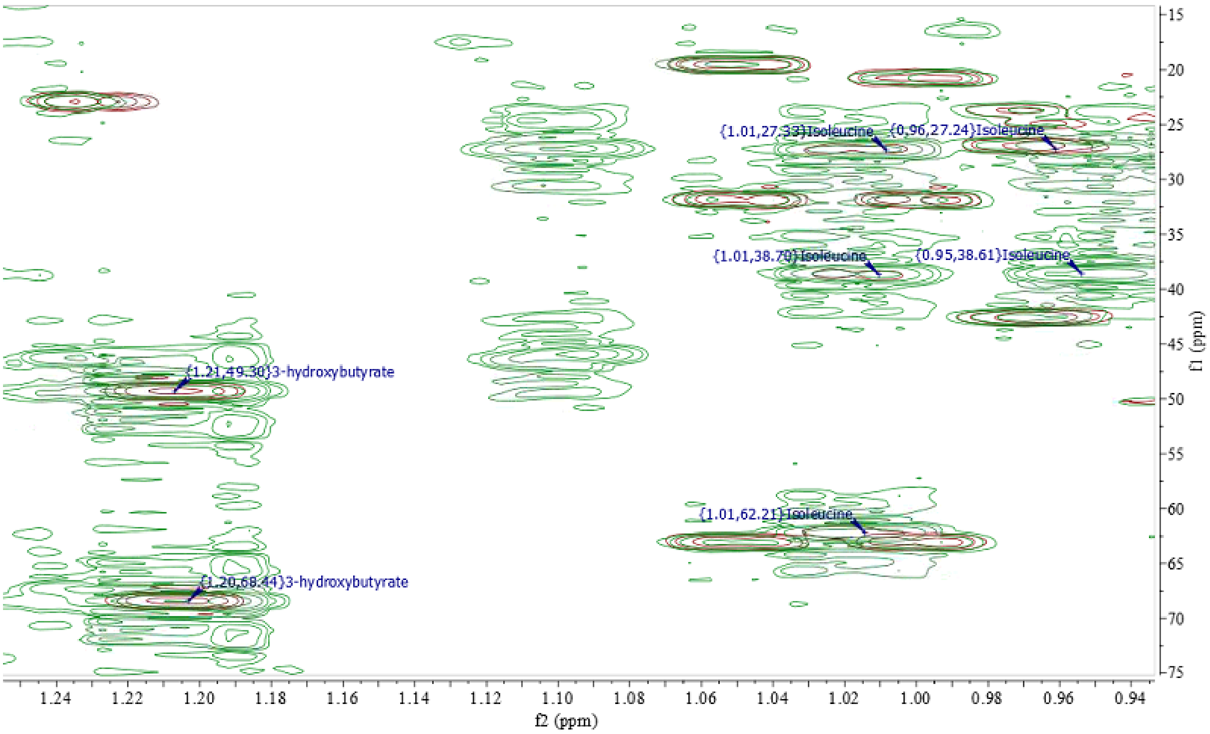

Supplementary Figure S9. Superimposition of the gHMBCad spectra of the reference sample before spiking (red circles) and after spiking (green circles) with isoleucine and 3-hydroxybutyrate.

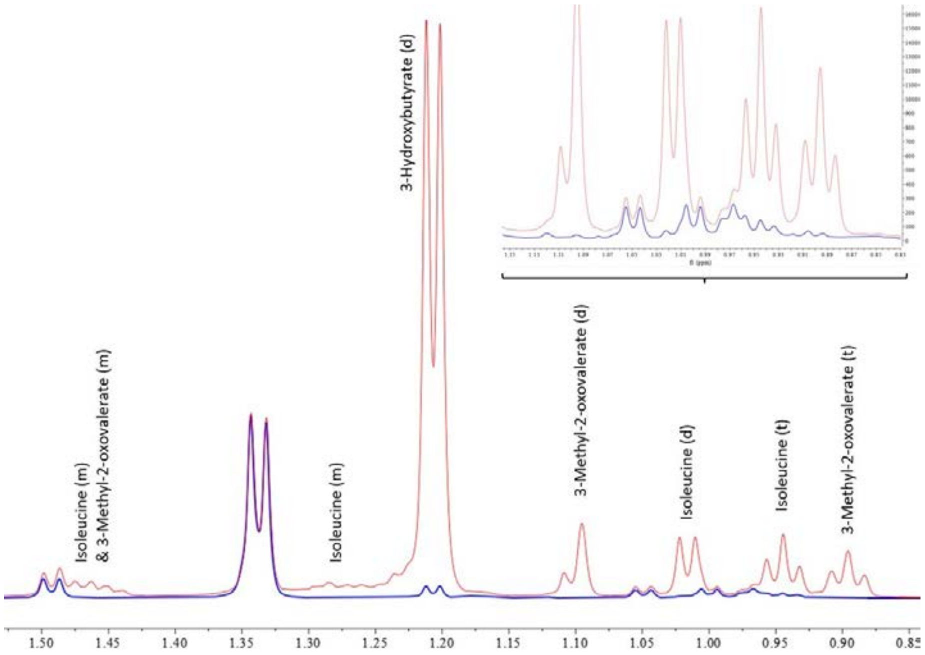

Supplementary Figure S10. Titration of the pool sample with 3-Methyl-2-oxovalerate, Isoleucine and 3-Hydroxybutyrate (red lines).

Supplementary Table S1. Identified metabolites, assignments and chemical shifts.

No	Metabolites	Assignments	Chemical Shifts-H
1	1-methylhistidine	CH_2 (2), CH_2 (5)	7.06 (s), 7.79 (s)
2	3-hydroxybutyrate	γ-CH_3, half α-CH_2, half α-CH_2, β-CH	1.20 (d), 2.31 (m), 2.41 (m), 4.15 (m)
3	3-methyl-2-oxovalerate	δ-CH_3, CH_3, half γ-CH_2, half γ-CH_2, β-CH	0.91 (t), 1.10 (d), 1.46 (m), 1.71 (m), 2.93 (m)
4	acetone	CH_3	2.24 (s)
5	acetylglycine	CH_3	2.04 (s)
6	alanine	β-CH_3, α-CH	1.48 (d), 3.78 (q)
7	arginine	half γ-CH_2, half γ-CH_2, β-CH_2, δ-CH_2, α-CH	1.67 (m), 1.75 (m), 1.91 (m), 3.24 (t), 3.76 (t)
8	asparagine	half β-CH_2, half β-CH_2, α-CH	2.85 (m), 2.95 (m), 4.00 (dd)
9	aspartate	half CH_2, half CH_2, CH	2.68 (dd), 2.82 (dd), 3.90 (dd)
10	betaine	CH_3	3.27 (s)
11	carnitine	CH_3	3.23 (s)
12	cholesterol	C18-CH_3	0.73 (m)
13	choline	CH_3, NCH_2, OCH_2	3.21 (s), 3.53 (m), 4.07 (m)
14	cis-aconitate	CH	5.68 (s)
15	citrate	half CH_2, half CH_2	2.55 (d), 2.67 (d)
16	creatine	CH_3, CH_2	3.04 (s), 3.93 (s)
17	creatinine	CH_3, CH_2	3.05 (s), 4.07 (s)
18	cysteine	CH_2, CH	3.05 (d), 3.95 (t)
19	cystine	half β-CH_2, half β-CH_2, α-CH	3.18 (dd), 3.38 (dd), 4.09 (dd)
20	dimethylglycine	CH_3	2.93 (s)
21	ethanol	CH_3, CH_2	1.17 (t), 3.65 (q)

22	ethanolamine	NCH_2, OCH_2	3.15 (t), 3.83 (t)
23	formate	CH	8.46 (s)
24	glutamate	half β-CH_2, half β-CH_2, γ-CH_2, α-CH	2.06 (m), 2.11 (m), 2.36 (m), 3.77 (dd)
25	glutamine	β-CH_2, γ-CH_2, α-CH	2.14 (m), 2.46 (m), 3.78 (t)
26	glycerol	half CH_2, half CH_2, CH	3.55 (dd), 3.66(dd), 3.78 (m)
27	glycerophosphocholine	CH_3, NCH_2, OCH_2CH_2	3.22 (s), 3.68 (t), 4.32 (t)
28	glycine	CH_2	3.56 (s)
29	hippurate	$C_{3,5}H$, $C_{2,6}H$	7.54 (m), 7.83 (dd)
30	histidine	half β-CH_2, half β-CH_2, α-CH,<br>NCHC, NCHNH	3.12 (dd), 3.23(dd), 3.99 (dd),<br>7.07 (s), 7.82 (s)
31	inosine	CH_2, -O-C**H**-CH_2OH, -C**H**(OH)-CH-,<br>-N-CH-, N-CH-N, -N-CH-N (imid.)	3.85 (d), 4.28 (m), 4.44 (dd),<br>6.10 (d), 8.24 (s), 8.34 (s)
32	isoleucine	δ-CH_3, CH_3, half γ-CH_2, half γ-CH_2,<br>β-CH, α-CH	0.94 (t), 1.01 (d), 1.24 (m), 1.46 (m),<br>1.97 (m) 3.69 (m)
33	lactate	β-CH_3, α-CH	1.33 (d), 4.11 (q)
34	leucine	δ-CH_3, δ-CH_3, γ-CH & β-CH_2, α-CH	0.96 (d), 0.97 (d), 1.69-1.74 (m), 3.74 (m)
35	lipids /<br>unsaturated lipid chains	CH_3, -(CH_2)n /<br>various CH, various CH_2	0.82 (m), 1.16 (m) /<br>2.77 (m), 5.30 (m), 5.35 (m)
36	lysine	half γ-CH_2, half γ-CH_2, δ-CH_2, β-CH_2,<br>ε-CH_2, α-CH	1.45 (m), 1.52(m), 1.73 (m), 1.91 (m),<br>3.03 (t), 3.76 (t)
37	malate	half CH_2, half CH_2, CH	2.38 (dd), 2.67 (dd), 4.30 (dd)
38	methanol	CH_3	3.36 (s)
39	methionine	β-CH_2, γ-CH_2	2.16 (m), 2.63 (t)
40	myo-inositol	C_2H, $C_{4,6}H$, $C_{1,3}H$	3.28 (t), 3.52 (dd), 3.61 (t)

41	ornithine	γ-CH_2, β-CH_2, δ-CH_2, α-CH	1.75 (m), 1.94 (m), 3.06 (t), 3.77 (t)
42	phenylalanine	half β-CH_2, half β-CH_2, α-CH,<br>δ-CH ring, στ-CH ring, ε-CH ring	3.14 (dd), 3.28 (dd), 4.00 (dd),<br>7.33 (m), 7.38 (t), 7.43 (m)
43	proline	γ-CH_2, half β-CH_2, half β-CH_2,<br>half δ-CH_2, half δ-CH_2, α-CH	1.99 (m), 2.05 (m), 2.35 (m),<br>3.34 (m), 3.41 (m), 4.14 (dd)
44	propylene glycol	CH_3, half-CH_2, half-CH_2,	1.15 (d), 3.39 (dd), 3.54 (dd)
45	pyroglutamate	half β-CH_2, γ-CH_2, half β-CH_2, α-CH	2.03 (m), 2.41 (m), 2.51(m), 4.17 (dd)
46	serine	α-CH, β-CH_2	3.85 (dd), 3.95 (m)
47	sucrose	CH	5.40 (d)
48	sugar chains	various CH,<br><br>various **CH_2**	3.27-3.32 (m), 3.42(m), 3.44 - 3.52 (m), 3.55 (m),<br>3.58-3.65 (m), 3.72 (m), 3.74 (m), 3.83(m),<br>**3.80-4.0 (m)**
49	taurine	SCH_2, CH_2	3.26 (t), 3.42 (t)
50	threonine	γ-CH_3, α-CH, β-CH	1.33 (d), 3.59 (d), 4.25 (m)
51	tryptophan	half β-CH_2, half β-CH_2, α-CH, CH ring (8),<br>CH ring (9), CH ring (6), CH ring (7)	3.32 (dd), 3.49 (dd), 4.06 (dd), 7.21 (t),<br>7.30 (t), 7.55 (d), 7.74 (d)
52	tyrosine	half CH_2, half CH_2, α-CH,<br>ε-CH ring, δ-CH ring	3.07 (dd), 3.20 (dd), 3.94 (dd),<br>6.90 (m), 7.20 (m)
53	uridine	CH ring, CH ring	5.90 (d), 7.88 (d)
54	valine	γ-CH_3, γ-CH_3, β-CH, α-CH	0.99 (d), 1.05 (d), 2.27 (m), 3.61 (d)
55	α-mannose	CH	5.19 (d)
56	α-glucose	CH	5.24 (d)
57	β-alanine	α-CH_2, β-CH_2	2.56 (t), 3.19 (t)
58	β-glucose	CH	4.65 (d)

Supplementary Table S2. Pearson correlation coefficient, r, between the integrated areas of the remaining lipid peak at 0.82 ppm and the areas of the BCAAs and the 3-hydroxybutyrate of representative spectra at umbilical cord and maternal sample pairs.

**Metabolite**	**Correlation coefficient, r**	
	**Umbilical (5IUGR+5AGA)**	**Maternal (5IUGR+5AGA)**
Leucine	0.579	0.624
Isoleucine	0.505	0.624
Valine	0.638	0.492
3-Hydroxybutyrate	0.152	0.261

Supplementary Table S3. Pearson correlation coefficient, r, between the integrated areas of the remaining lipid peak at 1.16 ppm and the areas of the BCAAs and the 3-hydroxybutyrate of representative spectra at umbilical cord and maternal sample pairs.

**Metabolite**	**Correlation coefficient, r**	
	**Umbilical (5IUGR+5AGA)**	**Maternal (5IUGR+5AGA)**
Leucine	0.619	0.616
Isoleucine	0.549	0.446
Valine	0.310	0.544
3-Hydroxybutyrate	0.468	0.308

Supplementary Table S4. Enrichment analysis of umbilical cord blood sample pairs

Metabolite Set	Total	Hits	P value	Holm P	FDR
Valine, Leucine and Isoleucine Degradation	60	3	0.0057	0.555	0.555
Tryptophan Metabolism	60	2	0.0586	1.0	1.0
Glucose-Alanine Cycle	13	1	0.0858	1.0	1.0

Supplementary Table S5. Enrichment analysis of maternal blood sample pairs

Metabolite Set	Total	Hits	P value	Holm P	FDR
Valine, Leucine and Isoleucine Degradation	60	3	0.0057	0.555	0.555
Glucose-Alanine Cycle	13	1	0.0858	1.0	1.0
Alanine Metabolism	17	1	0.111	1.0	1.0

Supplementary Table S6. Clinical characteristics of participating pregnant women.

Sample	Class	Percentile	Birth weight (g)	Gender	Gestational age (days)	Smoking	Maternal Pathology	Delivery	Mother age	Nationality	Maternal BMI before delivery
1	IUGR	5	2550	Female	273	N	N	Vaginal	32	Greek	19.3
2	IUGR	7	2930	Female	276	N	N	Caesarean	34	Romanian	24.1
3	IUGR	3	2850	Male	278	N	N	Vaginal	25	Greek	21.8
4	IUGR	1	1920	Female	265	Y	N	Caesarean	30	Greek	33
5	IUGR	4	2450	Female	271	N	N	Caesarean	26	Cypriot	19
6	IUGR	4	2560	Female	269	Y	N	Caesarean	36	Greek	23
7	IUGR	8	2880	Female	272	Y	N	Caesarean	31	Greek	33.4
8	IUGR	2	2230	Female	260	N	N	Vaginal	41	Greek	25
9	IUGR	2	2510	Male	275	N	N	Caesarean	31	Greek	25
10	IUGR	10	2760	Male	265	Y	N	Vaginal	30	Greek	25.7
11	IUGR	6	2690	Female	277	N	N	Vaginal	34	Greek	21
12	IUGR	5	2800	Female	277	N	N	Vaginal	26	Greek	26
13	IUGR	3	2540	Female	274	Y	N	Caesarean	27	Greek	19
14	IUGR	6	2450	Female	267	N	N	Caesarean	25	Georgian	23

15	IUGR	2	2860	Male	281	Y	N	Caesarean	33	Greek	28
16	IUGR	4	2770	Female	283	Y	N	Caesarean	36	Greek	34
17	IUGR	5	2250	Female	260	Y	N	Caesarean	30	Greek	33
18	IUGR	6	2850	Female	281	N	N	Vaginal	36	Greek	32
19	IUGR	10	2880	Male	274	N	N	Caesarean	35	Romanian	21
20	IUGR	8	2730	Female	279	N	N	Vaginal	28	Albanian	22
21	IUGR	4	2600	Male	273	N	N	Vaginal	34	Philippenese	21
22	IUGR	10	2560	Female	263	N	N	Vaginal	28	Greek	31
23	IUGR	10	2920	Male	274	N	N	Vaginal	38	Greek	26
24	IUGR	9	2780	Male	270	N	N	Caesarean	32	Albanian	24
25	IUGR	10	2590	Female	275	N	N	Vaginal	24	Albanian	20
26	IUGR	9	2590	Female	270	N	N	Vaginal	35	Greek	20
27	IUGR	3	2760	Female	279	N	N	Vaginal	29	Greek	26
28	IUGR	10	2160	Female	262	N	N	Caesarean	27	Albanian	23
29	IUGR	8	2840	Female	277	N	N	Caesarean	26	Albanian	26
30	IUGR	6	2880	Male	275	N	N	Vaginal	46	Albanian	25
31	IUGR	9	2780	Male	280	N	N	Caesarean	31	Greek	24
32	IUGR	8	2610	Female	273	N	N	Vaginal	32	Greek	21
33	IUGR	6	2220	Male	262	Y	N	Caesarean	26	Albanian	22

34	IUGR	1	1890	Male	260	N	N	Caesarean	41	Philippenese	25
35	IUGR	2	2200	Female	260	Y	N	Caesarean	40	Greek	22
36	IUGR	1	2430	Male	275	N	N	Caesarean	28	Greek	21
37	IUGR	3	2140	Female	260	Y	N	Caesarean	25	Albanian	20
38	IUGR	5	2650	Male	276	Y	N	Vaginal	35	Greek	20
39	IUGR	10	2650	Female	268	Y	N	Caesarean	45	Greek	20
40	IUGR	5	2760	Male	276	N	N	Vaginal	38	Greek	20
41	IUGR	7	2550	Female	261	N	N	Caesarean	30	Cypriot	22
42	IUGR	9	2920	Female	277	N	Gestational Diabetes Mellitus	Vaginal	32	Greek	19.6
43	IUGR	7	2820	Male	266	N	Gestational Diabetes Mellitus	Caesarean	37	Greek	23
44	IUGR	3	2590	Female	275	Y	Gestational Diabetes Mellitus	Caesarean	32	Greek	28
45	IUGR	2	2210	Female	263	Y	Gestational Diabetes	Caesarean	34	Greek	20

							Mellitus				
46	IUGR	7	2530	Female	264	N	Gestational Diabetes Mellitus	Caesarean	36	Russian	27
47	IUGR	3	2800	Male	282	N	Gestational Diabetes Mellitus	Caesarean	32	Romanian	27
48	IUGR	4	2510	Female	272	N	Thrombophilia	Caesarean	24	Albanian	21
1	AGA	54	3410	Male	275	N	N	Vaginal	35	Greek	22
2	AGA	40	3390	Male	280	N	N	Vaginal	40	Greek	20
3	AGA	42	3460	Male	285	N	N	Vaginal	35	Greek	23
4	AGA	40	3140	Female	274	N	N	Caesarean	24	Syrian	21
5	AGA	42	3390	Male	272	N	N	Caesarean	31	Greek	31
6	AGA	44	3490	Male	281	N	N	Vaginal	30	Moldovan	21
7	AGA	56	3180	Male	274	N	N	Vaginal	28	Greek	18
8	AGA	47	3520	Male	276	N	N	Vaginal	34	Georgian	22
9	AGA	65	3380	Female	274	N	N	Caesarean	34	Greek	25
10	AGA	43	3160	Female	280	N	N	Vaginal	37	Greek	19

11	AGA	40	3270	Male	273	N	N	Vaginal	27	Albanian	21
12	AGA	65	3000	Female	269	N	N	Vaginal	23	Greek	21
13	AGA	42	2930	Female	285	N	N	Vaginal	21	Albanian	17
14	AGA	52	3700	Male	282	N	N	Vaginal	25	Greek	34
15	AGA	65	3250	Female	269	N	N	Vaginal	30	Greek	24
16	AGA	52	3430	Female	277	N	N	Vaginal	32	Romanian	23
17	AGA	65	3740	Female	280	N	N	Caesarean	33	Greek	33
18	AGA	53	3610	Female	276	N	N	Vaginal	29	Greek	19
19	AGA	56	3410	Female	286	N	N	Vaginal	25	Albanian	19
20	AGA	44	2440	Female	261	N	N	Caesarean	31	Albanian	24
21	AGA	65	4000	Male	290	N	N	Caesarean	33	Greek	28
22	AGA	40	3250	Female	282	N	N	Vaginal	32	Romanian	21
23	AGA	42	3370	Male	275	N	N	Caesarean	48	Greek	29
24	AGA	41	3430	Female	283	N	N	Vaginal	40	Greek	23
25	AGA	42	3250	Female	275	N	N	Vaginal	38	Greek	22
26	AGA	60	3450	Female	278	N	N	Caesarean	40	Greek	22
27	AGA	54	3400	Female	283	N	N	Vaginal	29	Albanian	22
28	AGA	63	3490	Female	280	N	N	Vaginal	37	Greek	27
29	AGA	66	3590	Male	274	N	N	Caesarean	29	Albanian	26

30	AGA	62	3460	Female	274	N	N	Caesarean	33	Albanian	24
31	AGA	60	3800	Male	284	N	N	Vaginal	33	Albanian	23
32	AGA	51	3790	Male	283	N	N	Vaginal	27	Russian	36
33	AGA	57	3100	Male	278	N	N	Vaginal	28	Greek	35
34	AGA	35	3230	Female	278	N	N	Vaginal	42	Greek	23
35	AGA	48	3520	Male	273	N	N	Caesarean	39	Greek	32
36	AGA	45	3350	Female	282	N	N	Caesarean	39	Croatian	20
